# Physiological Benefits and Performance of Sea Water Ingestion for Athletes in Endurance Events: A Systematic Review

**DOI:** 10.3390/nu14214609

**Published:** 2022-11-02

**Authors:** Jerónimo Aragón-Vela, Olivia González-Acevedo, Julio Plaza-Diaz, Rafael A. Casuso, Jesús R. Huertas

**Affiliations:** 1 Department of Physiology, School of Pharmacy, University of Granada, Campus de Cartuja s/n, 18071 Granada, Spain; 2 Institute of Nutrition and Food Technology “José Mataix”, Biomedical Research Center, University of Granada, 18016 Granada, Spain; 3 Department of Physical Education and Sport, Faculty of Sport Sciences, University of Granada, 18071 Granada, Spain; 4 Department of Biochemistry and Molecular Biology II, School of Pharmacy, University of Granada, 18071 Granada, Spain; 5 Instituto de Investigación Biosanitaria IBS.GRANADA, Complejo Hospitalario Universitario de Granada, 18014 Granada, Spain; 6 Children’s Hospital of Eastern Ontario Research Institute, Ottawa, ON K1H 8L1, Canada; 7 Departamento de Ciencias de la Salud, Universidad Loyola Andalucía, 41704 Sevilla, Spain

**Keywords:** exercise physiology, endurance exercise, sweating, hydration

## Abstract

In different endurance events, athletes have limited access to fluid intake, such as ultra-endurance running. For this reason, it is necessary to establish an adequate hydration strategy for this type of long-duration sporting event. Indeed, it seems that the intake of seawater is a suitable hydration alternative to improve post-exercise recovery in this type of endurance event. This seawater is characterized by being a deep natural mineral water of moderate mineralization, which is usually extracted from a depth of about 700 m. Therefore, the aim of this systematic review is to evaluate the efficacy of seawater consumption in both performance and post-exercise recovery in long-duration sport events. A systematic and comprehensive literature search was performed in PubMed, Scopus, and Web of Science in September 2022. Initially, 8 out of 558 articles met the inclusion criteria. Among these eight studies, six were randomized clinical trials, and two were observational studies (one cross-sectional and one prospective study in well-conditioned student athletes). The results showed that deep sea water consumption accelerated the recovery of aerobic capacity and leg muscle capacity on running performance. In addition, the lactate production after the running exercise in seawater was significantly lower than in pure water. In conclusion, the present review demonstrates that seawater consumption could significantly improve the capacity of recovery after exercise.

## 1. Introduction

Nowadays, endurance sporting events are experiencing an increase in popularity, especially during the summer season [1]. This situation would be defined as a hostile environment where the body is subjected to a prolonged and demanding effort, resulting in a state of dehydration within minutes [2]. Indeed, in triathlon events, in the swimming and running stages, due to their dynamic nature, fluid intake is limited [3]. For this reason, an efficient hydration strategy must be established [4]. This is to avoid a significant negative impact on both aerobic performance [5] and athlete health [6] due to progressive dehydration combined with hyperthermia [7]. In addition, the amount of fluid consumed and its composition should be carefully considered. Indeed, the excessive consumption of liquids without the necessary composition can induce a state of hyponatremia [8] and rehydration with an electrolyte-enriched drink may reduce susceptibility to sudden painful involuntary contractions [9]. Therefore, both the volume of the rehydration fluid and its composition are critical for maintaining whole body fluid homeostasis. Currently, a hydration alternative that is gaining popularity in the sports community is the intake of deep seawater (DSW) [10]. This seawater is characterized by being a deep natural mineral water of moderate mineralization, which is usually extracted from a depth of about 700 m [11]. This is because deep-ocean mineral water contains components that could complement and increase human recovery following an exhaustive physical challenge [12].

Although water is sufficient to rehydrate after short-term acute effort, in the case of endurance events, it may be insufficient and should be supplemented with the consumption of some type of meal that restores the loss of electrolytes. Indeed, such long-duration events generate large sweat losses, which induce significant salt elimination. Therefore, a rehydration program should be undertaken to restore electrolyte losses, leading to adequate recovery [13]. Stasiule et al. [11] reported that ingestion of deep mineral water could accelerate the recovery of aerobic capacity and leg muscle power compared with the ingestion of water alone [11]. In addition, it has been shown that desalinated ocean mineral water, taken from 662 m below sea level, can substantially accelerate the recovery of aerobic capacity and lower-body muscle power after a prolonged bout of dehydrating exercise [12]. DSW is mainly characterized by a high percentage of minerals such as magnesium (Mg). Indeed, Mg deficiency may potentiate exercise-induced muscle damage and stress, as well as exacerbate inflammation through an increase in the secretion of pro-inflammatory cytokines [14,15,16]. However, the evidence for the beneficial effects of DSW is still limited. This is because it has not been thoroughly evaluated in endurance sports, such as the triathlon, with the compounding factor that it has limited access to liquids.

Several studies have shown that a marked state of mild to moderate hypohydration occurs during ultramarathon running competitions [17,18,19,20]. This might be because not enough fluid was ingested to compensate for sweat losses during exercise [21]. When combined with hyperthermia, this can cause cardiovascular instability, such as reduced plasma volume, cardiac filling, and stroke volume [7,22]. Consequently, dehydration may induce a stress response and, thus, suppress immune function [23,24,25], and even increase exercise-induced inflammatory responses [26]. Dehydration, in particular, can cause a systemic response of cytokine production and an imbalance between pro-inflammatory and anti-inflammatory cytokines in the system [23], which could negatively interfere with the regeneration of damaged tissue after exercise [22].

Therefore, it would be interesting to report how DSW consumption alters the inflammatory response and the muscular damage induced by exercise and its relationships with post-exercise recovery. In summary, the authors of this systematic review are interested in finding out if DSW consumption could provide an adequate hydration alternative, which could improve performance and recovery periods post-exercise. Therefore, the main purpose of the present systematic review was to evaluate the efficacy of a hydration strategy using DSW consumption, mainly in endurance events.

## 2. Materials and Methods

### 2.1. Search Strategy

Based on the Preferred Reporting Items for Systematic Reviews and Meta-Analyses (PRISMA) guidelines [27], this systematic review was conducted. An exhaustive bibliographic search was conducted in three databases (Web of Science, PubMed, and Scopus), from 1 January 2000 to 31 July 2022. As there has been no systematic review of seawater consumption in endurance events, we have set the starting year as 2000 in order to identify as many studies from the last 22 years as possible. A search strategy for Web of Science, PubMed, and Scopus is shown in Table 1. This systematic review was listed on the PROSPERO (International prospective register of systematic reviews) website on 9 July 2022, with the following record CRD42022341587. Available from: https://www.crd.york.ac.uk/prospero/display_record.php?ID=CRD42022341587 (accessed on 30 August 2022).

### 2.2. Criteria Used for Selection

In the selection process, the following criteria were used: (i) English-written articles; (ii) databases derived from Web of Science, PubMed, and Scopus; (iii) human studies; (iv) original articles: clinical trials; randomized controlled trials (RCTs); quasi-experimental studies; long-term, prospective, and cross-sectional studies; (v) articles published between January 2000 and July 2022. Exclusion criteria included: (i) studies that included persons suffering from pathologies; (ii) studies that included children under the age of 18 and older adults (+50 years); (iii) studies that included supplements or dietary interventions; (iv) case studies, case reports, letters to the editor, systematic reviews and meta-analyses, and narrative reviews. Training subjects were not restricted in terms of their body composition. Following the removal of duplicates, eligibility was determined by reading the title and abstract, and if still potentially eligible, by reading the full text.

### 2.3. Reliability and Extraction of Data

Three independent reviewers conducted the search (Jerónimo Aragón-Vela, Olivia González-Acevedo, and Julio Plaza-Diaz). The authors read the titles and abstracts of all the articles that were retrieved. In order to resolve disagreements regarding eligibility, a meeting was held. As part of the analysis of each included study, the following information was collected: the first author, the publication year, the type of study, the objective, the number of subjects, gender, age, when the information was available, type of exercise, how the exercise was performed, biochemical markers of fatigue, the inflammatory response, the variables in sports performance (exercise recovery periods, mainly in endurance events), and the main conclusions and findings of DSW consumption.

Based on the type of study (low, medium, or high intensity, or long-duration exercise interventions, and humans that consumed DSW), the selected articles were categorized. A review of the results of the studies that met the selection criteria for their recovery was conducted.

### 2.4. Evaluation of the Validity and Reliability of the Evidence

We assessed the risk of bias using the Joanna Briggs Institute’s Critical Appraisal Tool for Systematic Reviews developed by the Joanna Briggs Institute, Adelaide, Australia [28]. To summarize, this tool contains four specific checklists for different types of studies (i.e., cross-sectional, quasi-experimental, cohort, and RCTs studies). For each of them, there are four possible answers: “yes” (criterion met) and “no” (criterion not met). There were eight items for cross-sectional studies, nine items for quasi-experimental, and thirteen items for RCTs. Based on the above criteria, the studies are considered as “low-quality” evidence when ≤49% of the items are classified as “yes” (criterion met). As a result, articles are considered “medium-quality” evidence if 50–74% of the items are scored as “yes” and “high-quality” evidence when ≥75% of the items are scored as “yes”. The total percentage excludes the answers “not applicable” and “not clear” [29,30,31]. Each of the three reviewers assessed the quality of the studies separately. For the purpose of resolving possible differences between the reviewers, a consensus meeting was organized.

## 3. Results

**Figure 1:**
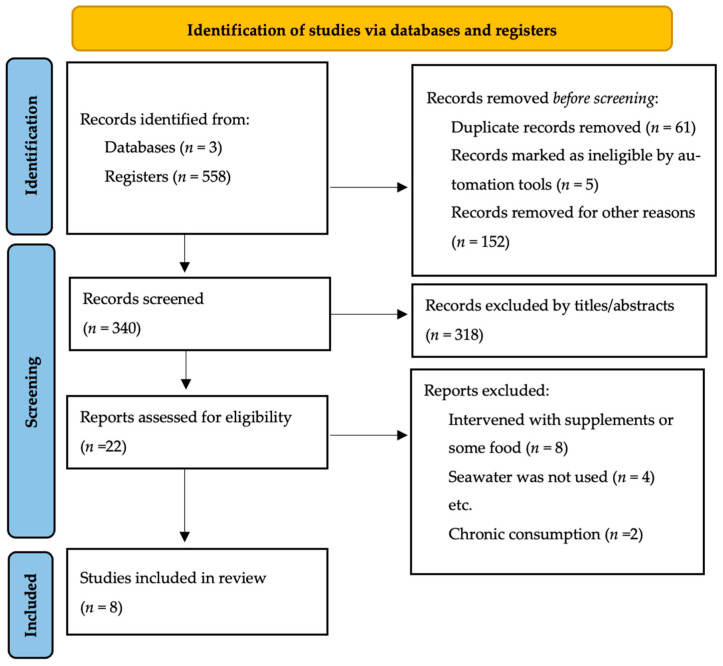
Flow chart systematic review [27].

A flow chart illustrating the selection of reporting elements for systematic reviews is depicted in Figure 1. The three databases assessed contained a total of 558 studies. Based on the title and abstract and duplicates, 195 studies were excluded, and 23 studies were excluded for being outside the scope of the review. The eligibility of 22 studies was assessed. Based on the exclusion criteria, eight studies were included in the analysis. There were six RCTs, and two observational studies (one cross-sectional and one well-conditioned student-athletes study).

The quality of the selected studies is summarized in Table 2. All studies were designed as a high-quality study according to the checklist from Joanna Briggs Institute’s criterium. Table 3 and Table 4 (measuring performance) summarize the main results of the selected articles. According to the information provided above, it appears that the majority of the studies are RCTs.

### 3.1. Selected Studies

Our selected investigations used different forms of DSW, and we divided the results according to that, starting with DOM, followed by deep mineral water (DMW), and seawater.

#### 3.1.1. Deep-Ocean Mineral Water

In response to exercise and heat, the body becomes dehydrated and the osmolality of extracellular fluid increases, which results in decreases in exercise performance and poor thermoregulation. It has been demonstrated in previous studies that DOM can promote the recovery of exercise performance following exercise [34].

This study evaluated the effects of DOM on recovery from a fatiguing exercise conducted at 30 °C using a randomized, double-blind, placebo-controlled crossover design. During the fatiguing exercise protocol, aerobic power was significantly reduced (reduced VO_2_max) for 48 h. In contrast, supplementation with DOM led to the complete recovery of aerobic power within four hours. It was also found that muscle power increased over placebo levels within 24 h of recovery. DOM completely eliminated elevated circulating creatine kinase and myoglobin, which are indicatives of exercise-induced muscle damage [12].

On the other hand, Wei et al., in their study, examined the effects of DOM supplementation on the cerebral hemodynamic response to physical exertion in young and middle-aged men. In this study, young and middle-aged men were subjected to double-blind placebo-controlled crossover studies [32]. There was a 2-week washout period between the counterbalanced trials of DOM and placebo. Before, during, and after cycling exercise, DOM and placebo were orally supplemented in beverages. This product contains desalinated minerals and trace elements extracted from seawater collected at a depth of 618 m. An infrared spectroscopy was used to measure the cerebral hemodynamic response (tissue hemoglobin) during cycling at a maximum oxygen consumption of 75%. Placebo and DOM trials for both age groups produced similar times to exhaustion at 75% VO_2_max and plasma lactate responses. A comparison of DOM with placebo showed significant increases in the levels of cerebral hemoglobin in young men and, to a greater extent, in middle-aged men. In middle-aged men, 2 h after exhaustive cycling, neutrophil-to-lymphocyte ratios were increased, but DOM attenuated these effects [32].

In order to achieve a 3% loss in body mass, subjects were exposed to an exercise-dehydration protocol (stationary biking) under warm conditions (30 °C). After exercise, the subjects were given DOM, mountain spring water, or a carbohydrate-based sports drink in a volume comparable to the loss of body mass. During exercise and after exercise rehydration, saliva samples were collected at regular intervals. The participants were also asked to perform peak torque knee extensions in order to assess the strength of their lower body muscles. When the subjects were given DOM during the rehydrating period, their hydration state returned to pre-exercise (baseline) more rapidly, as measured by the rate at which salivary osmolality dropped from peak to baseline, as compared to those receiving carbohydrate-based sports drinks and mountain spring water. Furthermore, following rehydration with DOM, subjects demonstrated significantly improved muscle recovery compared to those who received carbohydrate-based sports drinks or mountain spring water [10]. Based on a similar methodology, mountain spring water and carbohydrate-based sports drinks were compared for relative hydration and muscle strength using peak torque from leg extension maneuvers. During the dehydrating exercise protocol, salivary osmolality increased significantly with the loss of body mass. In comparison to females, males took less time to lose 3% of their body weight. A mono-exponential model was used to fit salivary osmolality during the rehydration phase. Both male and female participants receiving DOM as the hydrating fluid returned to baseline salivary osmolality most rapidly regardless of whether they fit stimulated or unstimulated salivary osmolality. There was a greater peak torque generated by males compared to females at baseline and immediately after a 3% loss in body mass. The rehydration fluid and sex also had a significant effect on peak torque following rehydration [34].

Supplementation with DOM was evaluated in a high-intensity intermittent running capacity following short-term recovery from a prolonged bout of high-intensity running in a thermoneutral environment. It was conducted using a double-blind, repeated measures, crossover, and counterbalanced design on nine healthy recreational male soccer players to determine their peak oxygen uptake, two familiarization trials, and two experimental trials. A seven-day interval separated each trial, and all trials were conducted at room temperature. The participants were provided with either DOM or a taste-matched placebo, mixed with 6% sucrose, during the 2 h recovery period after running at 75% peak oxygen uptake for 60 min. Comparatively to placebo, DOM increased the ability to run at high intensity by 25%. In terms of blood lactate concentration, blood glucose concentration, or urine osmolality, there were no differences between DOM and placebo [35].

#### 3.1.2. Deep Mineral Water

The effects of DMW with moderate mineralization on the recovery of physical performance during aerobic exercise in the heat were evaluated in a similar study using DMW with moderate mineralization. This study was a randomized, double-blind, placebo-controlled crossover of nine healthy and physically active women of various ages. As a result of rehydrating with DMW after 4 h, VO_2_max was 9% higher than that after rehydrating with plain water. After 48 h of rehydrating with DMW compared with plain water, leg muscle power recovered better during the slow phase of recovery [11].

#### 3.1.3. Sea Water

The purpose of Perez-Turpin et al.’s study was to assess the effects of ingestion of microfiltered and sterilized seawater on running performance in a hot environment [33]. The study consisted of 12 experienced male runners who participated in a crossover, double-blind randomized trial. A random sample of 50 mL of seawater or pure water was administered five minutes prior to running at 40% of their maximum oxygen consumption for ninety-five minutes at 30 °C until losing three percent of their body weight. The body weight of the subjects was measured every 20 min, and the blood lactate level was measured. As compared to the pure water condition, there was a significant decrease in lactate concentration following the running exercise in the seawater condition [33].

The purpose of this study was to assess the efficacy of seawater hydration on cytokine production during a triathlon. In this study, fifteen trained male triathletes were randomly assigned to participate in three triathlons, the first in which seawater was consumed (Totum SPORT, Laboratories Quinton International, S.L., Valencia, Spain), the second in which tap water was consumed ad libitum, and the third in which placebo (physiologic saline solution) was administered. The triathlon consisted of a swim of 800 m, a bike ride of 90 km, and a run of 10 km. After training and at rest, blood samples were collected to determine inflammation markers, hemoglobin concentration, and hematocrit concentration. However, the seawater did not increase performance, but it did increase the levels of interleukin (IL)-6 and apelin following exercise. In spite of this, there were no differences between the placebo group and the experimental group in terms of fractalkine, IL-15, erythropoietin, osteonectin, myostatin, oncostatin, irisin, follistatin-like 1, osteocrin, brain-derived neurotrophic factor, and fibroblast growth factor 21 levels [36].

## 4. Discussion

The main objective of the present systematic review was to evaluate the benefits of DSW consumption as a hydration strategy, mainly in endurance events. To our knowledge, this is the first systematic review that attempts to summarize the benefits of DSW consumption as a hydration alternative after endurance or strength events. Eventually, our results showed that DSW consumption could significantly improve functional recovery after exercise, as well as the inflammatory response.

DSW is regarded as a natural ocean resource due to the character of its low temperature, high purity, and mineral richness [37]. Several countries are represented in the analyzed studies, including Spain, Taiwan, Florida, and Arizona. Seawater samples, however, showed similar characteristics, as indicated by their composition. Minerals in this beverage are evenly distributed, with Mg and sodium (Na) being the most predominant minerals, followed by potassium (K) and calcium (Ca). Thus, we believe that these differences between countries should not result in significant metabolic changes that would bias the findings of the recent study.

DSW has been shown to improve both human and animal models of atherosclerosis, dermatitis, diabetes, obesity, hyperlipidemia, and hypertension [38]. According to our results in the field of athletic hydration, there seems to be a link between the consumption of DSW and an acceleration of recovery from physical fatigue. This may be due to the presence of abundant minerals, such as Mg, Ca, and K, as well as several beneficial trace elements such as chromium, selenium, zinc, and vanadium, which are known to enhance overall health [37,38,39]. Mg is present as a complex with ATP in the mitochondria. It is essential for the structural function of a number of mitochondrial proteins, including the mitochondrial electron transport chain complex subunits, and pyruvate dehydrogenase phosphatase [40]. However, studies on Mg-deficient cultured human cells showed that mitochondrial dysfunction may induce a decrease in antioxidant defenses [41]. On the other hand, Ca is a key regulator of mitochondrial function and acts as a cofactor in several mitochondrial enzymes, as well as stimulation of the ATP synthase [39]. Furthermore, an increase in cytosolic Ca leads to increases in *PGC-1α* expression and mitochondrial biogenesis in rats [42]. Accordingly, the ergogenic effects demonstrated in the various studies examined could be attributed to the consumption of these minerals, which would induce a healthy alteration at the mitochondrial level, which would speed up post-exercise recovery.

For athletes engaged in competitive sports, hydration is critical to performance, injury prevention, and recovery [43]. In order to prevent illness and improve performance, strength and conditioning coaches should understand an athlete’s hydration needs [43]. In order to achieve normal hydration within six hours following exercise, athletes must consume 150% of the weight lost [44]. Therefore, it is recommended that each pound of body weight lost during training be replaced by 20 to 24 ounces (600 to 720 mL) of fluids [44]. Fluids commonly used by athletes include water, sports drinks, and isotonic or carbohydrate drinks [45,46]. It has been demonstrated in some studies that when an experimental group consumed DOM [34] or seawater [36] instead of water and sport drinks, fatigue was significantly reduced. In addition, metabolic aspects were improved.

According to Ha et al., DSW consumption induces mitochondrial biogenesis activity (it promotes expression of *PGC1-α*, *NRF1*, and *TFAM* genes) in preadipocytes [39], where IL-6 plays a key role [47]. Acevedo et al. reported that seawater stimulates an early release of IL-6 post-exercise [36]. It is known that IL-6 has a canonical pro-inflammatory effect when secreted by macrophages, such as during chronic sedentarism [48]. However, if it is secreted during exercise, it can have an anti-inflammatory effect [49]. In addition, exercise-induced IL-6 could increase glucose uptake and mitochondrial content within skeletal muscle through modulation of AMP-activated protein kinase (AMPK) activity [47,50]. Notably, in vitro studies have also suggested that DSW can stimulate mitochondrial biogenesis through AMPK activation [40]. DSW might, therefore, promote skeletal muscle’s oxidative metabolism by exposing the muscle cells to IL-6 for a longer period of time [51]. However, this hypothesis needs to be tested further. Therefore, this increase in mitochondrial gene expression and enzyme activity could be the second reason why DSW consumption could accelerate post-exercise recovery.

**Figure 2:**
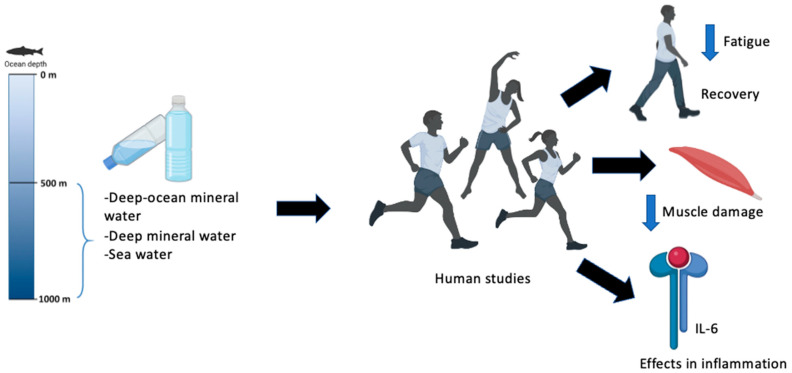
Main findings in the present systematic review.

Regarding the improvement of athletic performance, this finding is not very clear, as only a minority supports this hypothesis. Of the 10 studies analyzed, only 4 support it. Indeed, our current study showed no significant differences in athletic performance [36]. This discrepancy in the results obtained could be because no essential parameters of exercise intensity were taken into consideration. Thus, it is difficult to ensure that both hydration conditions were performed at the same relative intensity, as shown in the study by Pérez-Turpin et al., which did not use heart rate parameters that would have helped to ensure that both sessions guaranteed equal intensities [33]. However, in our study, heart rate parameters were used, which helped to have similar load intensities in both tests. The results of improving capacity are questionable. This is because, according to Higgins et al., only 4 of the 9 subjects improved their capacity with the consumption of DSW [35]. Two of them showed results that were double what they were compared to the placebo, which does not seem very convincing. Figure 2 summarizes the main findings of the present systematic review.

Based on the findings of the present systematic review, DSW consumption in human studies was associated with faster recovery from physical fatigue [12,33,34], improved systemic inflammation during recovery [32,36], and improved running capacity and performance [10,33,35].

### Limitations and Strength

There are a number of limitations to this study. A major limitation of this study is that the results were obtained from a limited number of human studies that are currently available in the literature. As a result, the data should be interpreted with caution. In addition, seawater consumption is still a novel therapy after this type of exercise, which may explain the small number of studies. (i) The selected studies were not all RCTs. There are, however, no studies that summarize the potential benefits of consuming DSW during sporting events, which is the main strength of the current review. Strength and endurance are examples of these. (ii) There have been some studies comparing DSW consumption with water and sport drinks; however, more RCTs are needed to verify this positive effect of DSW consumption.

## 5. Conclusions

With the main findings of the present systematic review, we can conclude that many of the studies reported that the consumption of DSW in endurance and strength exercises could help improve functional recovery postexercise in humans. It may even improve the inflammatory response, but more studies are needed to confirm this theory.

## Figures and Tables

**Table 1:** Search strategy in databases.

Database	Search Strategy	Limits	Filters
Web of Science	(ALL (deep sea water AND endurance exercise OR deep-sea water AND sweating OR deep-sea water AND sweating OR deep sea mineral water AND endurance exercise OR deep sea mineral water AND sweating OR deep sea mineral water AND hydration))	Publication date, 2000–2022, English language, Article, Search strategy (Topic)	54 items filtered
PubMed	(Deep sea water OR deep sea mineral water) AND (endurance exercise OR sweating OR hydration)	Publication date, 1 January 2000–31 July 2022, Humans, Adults: 18–50 years, English language, Search strategy (All Fields)	97 items filtered
Scopus	TITLE-ABS-KEY (deep AND sea AND water AND hydration) OR (deep AND sea AND water AND hydration AND endurance AND exercise) OR (deep AND sea AND water AND hydration AND endurance AND exercise AND barrier) OR (deep AND sea AND water AND hydration AND endurance AND exercise) AND LANGUAGE (English) AND (PUBYEAR > 2000)	Publication date, 2000–2022, English language, Article or review, Search strategy (TITLE-ABS-KEY)	407 items filtered

**Table 2:** Checklist from Joanna Briggs Institute’s criterium according to kind of study, percentage of criterium reached, and quality level of evidence.

Criteriums According to Kind of Study															
Authors	1	2	3	4	5	6	7	8	9	10	11	12	13	Percentage Reached	Quality Level
Hou et al., 2013 [12]	1	1	1	1	1	1	1	1	1	1	1	1	1	100%	HQ
Stasiule et al., 2014 [11]	1	1	1	1	0	0	1	1	1	1	1	1	1	85%	HQ
Keen et al., 2016 [10]	1	0	1	1	1	0	1	1						75%	HQ
Wei et al., 2017 [32]	0	1	1	1	1	1	1	1	1	1	1	1	1	92%	HQ
Pérez-Turpin et al., 2017 [33]	0	1	1	0	0	1	1	1	1	1	1	1	1	77%	HQ
Harris et al., 2019 [34]	1	1	1	1	1	1	1	1	1	1	1	1	1	100%	HQ
Higgins et al., 2019 [35]	1	1	1	1	1	1	1	1	1	1	1	1	0	92%	HQ
González Acevedo et al., 2022 [36]	0	1	0	1	1	1	1	1	1	1	1	0	1	76%	HQ

HQ: high quality. The values 1 and 0 indicate whether the item was achieved or not, respectively.

**Table 3:** Characteristics such as the type of study, aim, sample, design, mean results, and composition of the seawater of the studies.

First Author, Year of Publication	Country	Type of Study	Aim	Sample	Study Design	Results	Composition of the Seawater
Hou et al., 2013 [12]	Taiwan	Randomized, double-blind, placebo-controlled crossover study	DSW on recovery time	12 healthy, male volunteers. 24 ± 0.8 years.	The study was to evaluate the effect of DOM on the recovery time at 30 °C.	- The desalinated DOM can substantially accelerate recovery from physical fatigue in aerobic capacity (*p* < 0.05).	Following the two filtration procedures, molecules larger than 1.5 kilodalton were removed. As a means of masking the taste difference between DOM and placebo, the same amounts of sucrose, artificial flavors, citrate, citrus juice, calcium lactate, potassium chloride, vitamin C, and mixed amino acids were added to both.
Stasiule et al., 2014 [11]	Lithuania	Randomized, double-blind, placebo-controlled crossover study	The effect of DMW on the recovery	9 healthy, physically active, women. 24.0 ± 3.7 years.	Aerobic capacity (VO_2_ max) and peak lower-body muscle capacity were the measures selected for assessing the degree of recovery. VO_2_ max was measured using the ramp exercise test.	- Ingestion of DMW accelerated recovery of aerobic capacity (*p* < 0.05). - In comparison to water alone, DMW consumption increases the recovery of leg muscle capacity (*p* < 0.05).	Na (76 mg L^−1^), K (19 mg L^−1^), Ca (220 mg L^−1^), Mg (73 mg L^−1^), Cl (46 mg L^−1^), SO_4_ (874 mg L^−1^), F (0.3 mg L^−1^), Cu (0.0054 mg L^−1^), Fe (1.2326 mg L^−1^), Mn (0.0163 mg L^−1^), Cr (0.0025 mg L^−1^) P (0.5434 mg L^−1^), B (0.4175 mg L^−1^), and Zn (0.0124 mg L^−1^)
Keen et al., 2016 [10]	Arizona	Well-conditioned student-athletes study	DSW on rehydration	Eight student-athletes. Three experimental groups. 23.0 ± 1.2 years.	Subjects were exposed to an exercise-challenge under warm conditions. Body mass measurements were taken prior to exercise and then at 15 min intervals throughout the exercise trial and during rehydration. At each interval also, stimulated saliva was collected, and salivary osmolality was measured.	- Recovery of exercise performance post-exercise (*p* < 0.05).	Na (85 mg L^−1^), K (4 mg L^−1^), Ca (1.4 mg L^−1^), Mg (4.3 mg L^−1^), Cl (150 mg L^−1^), B (0.65 mg L^−1^), Bromide (540 µg L^−1^), and Cr (2.2 µg L^−1^)
Wei et al., 2016 [32]	Taiwan	Double-blind placebo-controlled crossover study	DOM supplementation on the cerebral hemodynamic response	9 middle-aged men and 12 young men. 46.8 ± 1.4, 21.2 ± 0.4 years, respectively	The counter-balanced trials of DOM and placebo were separated by a 2-week washout period. DOM and placebo were orally supplemented in drinks before, during, and after cycling exercise. Cerebral hemodynamic response was measured during cycling at 75% VO_2_ max using near-infrared spectroscopy.	- The DOM increased cerebral hemodynamic response (*p* < 0.05). - Reduced systemic inflammation during recovery (*p* < 0.05).	After this two-filtration procedure, molecules larger than 1.5 kilodalton were removed. For the purpose of masking the taste difference between DOM and placebo, the same amount of erythritol (3%) was added to each drink.
Harris et al., 2019 [34]	Arizona	Counterbalanced, crossover study	DOM supplementation on muscle performance recovery	17 participants. 20–25 years.	A dehydrating exercise protocol under heat stress until achieving 3% body mass loss. Participants rehydrated with either DOM water (Deep), mountain spring water (Spring), or a carbohydrate-based sports drink (Sports) at a volume equal to the volume of fluid loss.	- The DOM water positively affected hydration recovery after exercise (*p* < 0.0001). - It may also be beneficial for muscle strength recovery (*p* = 0.0117).	Na (85 mg L^−1^), K (4 mg L^−1^), Ca (1.4 mg L^−1^), Mg (4.3 mg L^−1^), Cl (150 mg L^−1^), B (0.65 mg L^−1^), Bromide (540 µg L^−1^), and Cr (2.2 µg L^−1^)
González Acevedo et al., 2022 [36]	Spain	CRS	SW and recovery	15 triathletes. 38.8 ± 5.6 years	Fifteen trained male triathletes randomly performed 3 triathlons, one of them consuming SW, the other one consuming tap water-hydration at libitum, and the last one with isocaloric placebo	- Interleukin-6 (*p* < 0.001) and apelin (*p* < 0.001) closely related to oxidative metabolism are potentially released by SW after triathlon event.	Electrolytes were supplied in the amount of 27.297 mg L^−1^ Na, 0.465 mg L^−1^ K, 19.5 mg L^−1^ Mg, and 1.377 mg L^−1^ Ca.

Abbreviations: CRS; crossover randomized study, DSW; deep sea water, DMW; deep mineral water, DOM; deep ocean mineral, SW; seawater.

**Table 4:** Characteristics such as the type of study, aim, sample, design, mean results, and composition of the seawater of the studies with performance.

First Author, Year of Publication	Country	Type of Study	Aim	Sample	Study Design	Results	Composition of the Seawater
Hou et al., 2013 [12]	Taiwan	Randomized, double-blind, placebo-controlled crossover study	DSW on performance	12 healthy, male volunteers. 24 ± 0.8 years	The study was to evaluate the effect of DOM on the exercise performed at 30 °C.	- Enhanced lower-body muscle capacity after a prolonged bout of dehydrating exercise (*p* < 0.05).	Following the two filtration procedures, molecules larger than 1.5 kilodalton were removed. As a means of masking the taste difference between DOM and placebo, the same amounts of sucrose, artificial flavors, citrate, citrus juice, calcium lactate, potassium chloride, vitamin C, and mixed amino acids were added to both.
Keen et al., 2016 [10]	Arizona	Well-conditioned student-athletes randomized study	DSW on physical performance	Eight student-athletes. Three experimental groups. 23.0 ± 1.2 years.	Subjects were exposed to an exercise-challenge under warm conditions. Body mass measurements were taken prior to exercise and then at 15 min intervals throughout the exercise trial and during rehydration. At each interval also, stimulated saliva was collected and salivary osmolality was measured.	- The DOM optimized exercise performance (*p* < 0.05).	Na (85 mg L^−1^), K (4 mg L^−1^), Ca (1.4 mg L^−1^), Mg (4.3 mg L^−1^), Cl (150 mg L^−1^), B (0.65 mg L^−1^), bromide (540 µg L^−1^), and Cr (2.2 µg L^−1^)
Pérez-Turpin et al., 2017 [33]	Spain	Crossover, double-blind randomized trial	SW ingestion on running performance	12 experienced male runners. 32 ± 6.4 years	Five minutes before the exercise protocol started, the subjects consumed SW or pure water in an equivalent amount of 50 mL. Subjects were asked to run on a motorized treadmill at 40% VO_2_ max at a room temperature of 30 °C until attaining a 3% decrease in body mass.	- The ingestion of microfiltered and sterilized seawater improved running performance and lactate production (*p* < 0.05).	Electrolytes were supplied in the amount of 27.297 mg L^−1^ Na, 0.465 mg L^−1^ K, 19.5 mg L^−1^ Mg, and 1.377 mg L^−1^ Ca.
Higgins et al., 2019 [35]	Taiwan	Crossover, and counterbalanced study	DOM supplementation on high-intensity intermittent running capacity	9 healthy, nonelite male soccer players. 22.0 ± 1.0 years	Two experimental trials were completed on a motorised treadmill, separated by 7 d, undertaken at ambient room temperature, and commenced at similar times of day. Incremental increases of 1 km h^−1^ were applied every three minutes until volitional exhaustion.	- DOM increased high-intensity intermittent running capacity in soccer players after short-term recovery from an initial bout of prolonged high-intensity running (*p* = 0.038).	Na (54.64 mg L^−1^), K (56.64 mg L^−1^), Ca (0.22 mg L^−1^), Mg (170.7 mg L^−1^), B (0.54 mg L^−1^), and Rb (0.02 mg L^−1^)

Abbreviations: CRS; crossover randomized study, DSW; deep sea water, DMW; deep mineral water, DOM; deep ocean mineral, SW; seawater.

## Data Availability

Not applicable.
